# Social determinants of youth with mild intellectual disability in outpatient care for mental health disorders: a case–control study

**DOI:** 10.1007/s00787-025-02718-5

**Published:** 2025-04-19

**Authors:** M. M. C. Storm, E. J. Giltay, W. M. van Eldik, E. C. Palstra, E. D. A. van Duin, D. van den Berg, R. R. J. M. Vermeiren

**Affiliations:** 1 LUMC Curium – Department of Child and Adolescent Psychiatry, Leiden University Medical Center, 2300 AA Leiden, The Netherlands; 2 Parnassia Group, Youz, De Banjaard, The Hague, The Netherlands; 3 Department of Psychiatry, Leiden University Medical Center, Leiden, The Netherlands; 4 Department of Clinical Psychology, VU University and Amsterdam Public Health Research, Amsterdam, The Netherlands; 5 Department of Psychosis Research, Parnassia Psychiatric Institute, The Hague, The Netherlands; 6 Department of Public Health and Primary Care, Health Campus The Hague, Leiden University Medical Center, The Hague, The Netherlands; 7 Youz, Parnassia Groep, The Hague, The Netherlands

**Keywords:** Intellectual disability, Mental health, Children in care, Social environment, Multivariate analysis

## Abstract

This study examined the unique role of multiple social determinants of mental health (SDOMH) associated with mental health disorders (MHD) for children with mild intellectual disability (MID), advancing understanding in a fragmented research area. Using a population-based case–control study design, four groups aged 0–17 years (*M*_age_ = 10.6, 35.6% female) were studied: children receiving outpatient mental health care for MHD with MID (*n* = 505) and without MID (*n* = 2767), each with a matched control group from the general population (*n* = 2525 and *n* = 13,835, respectively). Through multivariate logistic regression analyses, both MHD groups were compared to their control group and each other to examine the likelihood of a SDOMH associated with receiving mental health care for MHD in children with and without MID. Children with MID receiving mental health care showed significant differences in multiple domains compared to their control group and to children receiving mental health care without MID. They were less likely to have European-born mothers, more likely to have parents with moderate or low education levels, and tended to live in smaller, single-parent, lower-income households. Similar, though less deviant, patterns were observed for children receiving mental health care without MID compared to the general population, except for parental education. Our study highlights that SDOMH are associated with the likelihood of receiving care for MHD in children. Moreover, children with MID face disproportionate disadvantages, particularly regarding low parental education and household income. Thus, interventions should not only target the child but also their family and environmental context.

## Introduction

Children with mild intellectual disability (MID) face specific challenges threatening their development, particularly their mental health. Research consistently highlights the increased risk of psychopathology among children with MID compared to peers without MID [1–3]. In the socio-cultural, systemic paradigm, this heightened susceptibility is theorized to be shaped by a complex interplay of socio-demographic factors experienced by these children [4]. These factors, collectively known as social determinants of mental health (SDOMH), include ethnicity, socioeconomic status, household conditions, family dynamics, and neighborhood deprivation [5–7]. Although research has examined some SDOMH individually in relation to psychopathology in children with MID [8–11], the field remains fragmented. A critical gap persists in understanding the relative importance and collective role of multiple SDOMH in shaping mental health disorders (MHD) in this population. By identifying key SDOMH that are collectively associated with MHD in children with MID and comparing these findings with relevant groups, we aim to assess which SDOMH play a unique role for this population.

The few studies investigating the impact of SDOMH on children with MID reveal a concerning double burden. First, these children are vulnerable to experience environmental disadvantages, increasing their susceptibility to a lower social stratum [12, 13]. This, in turn, exposes them to higher levels of cumulative stress, stigmatization, and discrimination, which increases the risk of developing mental disorders [14]. Secondly, children with MID frequently face restricted access to vital resources such as wealth, social support, and problem-solving skills—key contributors to resilience in the face of adversity [7, 15]. Consequently, it can be hypothesized that adverse SDOMH will be more prevalent in children with both MID and MHD compared to their peers without MID [14, 16]. Testing this hypothesis and improving our understanding of the roles of different SDOMH will help address the unique challenges faced by children with MID and MHD.

To achieve this, we will organize SDOMH across domains using the conceptual framework of Lund et al. [17]. Each domain in this framework—demographic, social and cultural, economic, neighborhood, and environmental events—is hypothesized to be associated with mental health through various pathways. However, the last domain, encompassing factors such as war and natural disasters, is less common in the Netherlands and thus will not be addressed. This framework, initially developed for adults, will be extended to include SDOMH at parental and household levels for children.

Reviewing prior empirical studies in light of this framework, it becomes evident that demographic factors, particularly ethnicity, have been extensively studied as a variable associated with MHD among children with MID [18]. Although some studies found no link between ethnicity and MHD [10, 19], a systematic review of healthcare for minority ethnic groups with MID in the UK and findings from a scoping review indicate that current conclusions might be due to the potential underrepresentation of minority groups in both health services and research samples in general [20, 21]. Second, the social and cultural domain encompasses frequently studied family and parental characteristics. For instance, studies on the impact of a single-parent household on behavior problems yielded divergent findings; some studies reported no significant association [22, 23], others showed associations between single parenthood and higher levels of behavior problems [1, 4, 24]. Regarding economic factors, two systematic reviews came to the similar conclusion that associations between socioeconomic status (SES) and MHD were far from definite [2, 11]. Some studies found an association between lower SES and more MHD for children with MID [4, 25], whereas others reported no significant association [10, 26]. Importantly, some studies did not specify a method or criteria used for measuring SES, leaving the relative roles of different aspects of SES as contributors to MHD poorly understood [26]. Finally, few studies have explored neighborhood factors in relation to children with MID [9, 27], despite the generally well-documented impact of neighborhood deprivation on young people’s mental health and well-being [28].

This study aims to deepen our understanding of the associations between SDOMH and MHD in children with MID by comparing four groups: children with MID receiving mental health care, children without MID receiving mental health care, and their respective matched control groups from the general population. Additionally, we compare children without MID receiving mental health care to their matched control group from the general population. While some SDOMH have been individually examined in relation to MHD in both children with and without MID, a comprehensive analysis of their unique or combined effects is lacking. Comparing these associations across groups will identify key SDOMH related to MHD in children with MID. We hypothesize that adverse SDOMH, such as lower parental education, lower household income, and single-parent households, will be more prevalent in the group of children with both MID and MHD compared to children without MID receiving mental health care and the matched control group. Enhancing our understanding of the roles of SDOMH will help address the unique challenges faced by both children with and without MID who experience MHD, and inform preventive policy and intervention strategies to better support their mental health.

## Methods

### Sources of data

The study used data from the Extramural LUMC Academic Network data warehouse, a comprehensive regional population-based data infrastructure [29]. Three primary data sources were analyzed: microdata from Statistics Netherlands (SN; the central register agency), The Hague municipality data, and patient data from specialized youth mental health care institutions, including one for children with MID and MHD.

SN data provides insights into Dutch society, economy, and environment, comprising longitudinal microdata on demographics and socio-economic indicators [30]. The Hague municipality data provided neighborhood-specific information on urbanization, income, and education levels. Patient data included variables such as age, sex, and (un)registration in mental health care institutions.

### Participants

Participants were categorized into four groups: two clinical case groups and two matched population-based control groups. The clinical case groups were receiving outpatient treatment for MHD at a specialized facility and resided in The Hague or its surrounding areas. One group consisted of children diagnosed with MID (IQ 55–85) and MHD (Group A: MID + MHD) and the second included those with MHD, but without MID (Group B: MPH only). Participants were identified from patient data recorded in 2017. We used receipt of outpatient mental health care as a proxy for identifying children with MHD. Children were included if they were 0 to 17 years old and received outpatient care in 2017 at a participating mental health facility. Group A was identified from a mental health care facility specialized in the psychiatric assessment and treatment, including cognitive behavioral therapy and pharmacotherapy, of children with MID (*n*_A_ = 505). Group B was identified from registrations at other participating centers that provide mental health care for children without MID (*n*_B_ = 2767). All participants resided in urban or suburban areas, as this was a selection criterion based on the predominantly urban setting of The Hague and its surroundings. The year 2017 was chosen for its availability of essential demographic and patient data. Only outpatient children were included to explore family-related characteristics, as they resided at home.

Thereafter, two control groups were formed for Group A and B, comprising children from the general population matched by sex and age using the SN database: Group C1 (*n*_C1_ = 2525) for Group A and Group C2 (*n*_C2_ = 13,835) for Group B. Five controls were selected per case, residing in the same areas, with randomized postal codes for residential comparison. Baseline characteristics for all groups are presented in Table 1.

**Table 1:** Baseline characteristics by group

	Total sample N (%) N = 19,632	Group A: MID + MHD N = 505	Group C1: Controls N = 2525	Group B: MHD-only N = 2767	Group C2: Controls N = 13,835
**Demographic domain**					
Child sex:	19,632 (100%)	–	–	–	–
- Female	–	180 (35.6)	900 (35.6)	1231 (44.5)	6155 (44.5)
- Male	–	325 (64.4)	1625 (64.4)	1536 (55.5)	7680 (55.5)
Age child (years)	19,632 (100%)	10.6 (4.1)	10.6 (4.1)	12.0 (3.9)	12.0 (3.9)
Child’s country of birth:	19,632 (100%)	–	–	–	–
- The Netherlands	–	462 (91.5)	2301 (91.1)	2443 (88.3)	12,677 (91.6)
- EU (excluding NL)	–	20 (4.0)	102 (4.1)	137 (5.0)	511 (3.7)
- Outside EU	–	23 (4.5)	122 (4.8)	187 (6.7)	647 (4.7)
Age mother (years)	19,438 (99.0%)	40.1 (7.1)	41.6 (6.4)	42.0 (6.9)	43.0 (6.4)
Mother’s country of birth:	19,632 (100%)	–	–	–	–
- The Netherlands	–	296 (58.6)	1574 (62.3)	1761 (63.6)	8815 (63.7)
- EU (excluding NL)	–	43 (8.5)	203 (8.1)	216 (7.8)	1064 (7.7)
- Outside EU	–	166 (32.9)	748 (29.6)	790 (28.6)	3956 (28.6)
Age father (years)	18,321 (93.3%)	44.2 (7.9)	45.0 (7.2)	45.9 (7.6)	46.4 (7.0)
Father’s country of birth:	19,632 (100%)	–	–	–	–
- The Netherlands	–	281 (55.6)	1589 (62.9)	1730 (62.5)	8890 (64.3)
- EU (excluding NL)	–	32 (6.4)	164 (6.5)	188 (6.8)	836 (6.0)
- Outside EU	–	192 (38.0)	772 (30.6)	849 (30.7)	4109 (29.7)
**Social and cultural domain**					
Number of children (in household)	19,618 (99.9%)	2.0 (1.1)	2.3 (1.0)	2.0 (1.0)	2.3 (1.0)
Family type:	19,618 (99.9%)	–	–	–	–
- Dual parent household	–	274 (54.3)	1997 (79.1)	1550 (56.0)	10,857 (78.5)
- Single parent household	–	187 (37.0)	500 (19.8)	1055 (38.1)	2825 (20.4)
- Institutional household/other	–	44 (8.7)	28 (1.1)	162 (5.9)	153 (1.1)
Education level mother:	13,566 (69.1%)	–	–	–	–
- High	–	46 (12.0)	804 (45.3)	643 (32.4)	4199 (44.6)
- Moderate	–	165 (43.1)	523 (29.5)	681 (34.3)	2735 (29.0)
- Low	–	172 (44.9)	446 (25.2)	781 (33.3)	2490 (26.4)
Education level father:	11,199 (57.0%)	–	–	–	–
- High	–	38 (14.8)	759 (50.5)	539 (37.3)	4020 (50.3)
- Moderate	–	104 (40.5)	380 (25.3)	442 (30.6)	2069 (25.9)
- Low	–	115 (44.7)	364 (24.2)	464 (32.1)	1905 (23.8)
**Economic domain**					
Household income (in percentiles)	19,296 (98.3%)	33.5 (24.2)	52.0 (30.5)	42.6 (28.8)	53.7 (30.1)
**Neighborhood domain**					
Urbanization class:	19,632 (100%)	–	–	–	–
- Densely populated (class 1–3)	–	463 (91.7)	2108 (83.5)	2,473 (89.4)	11,374 (82.2)
- Sparsely populated (class 4, 5)	–	42 (8.3)	417 (16.5)	294 (10.6)	2461 (17.8)
% low neighborhood education level	14,456 (73.6%)	36.3 (12.8)	31.3 (14.1)	33.0 (12.9)	31.4 (14.2)
Low median neighborhood income (in percentiles)	9733 (49.6%)	15.0 (7.6)	12.9 (7.7)	13.2 (7.5)	13.1 (7.8)

For continuous variables, the mean and SD are provided (SD in brackets). For categorical variables, the N and percentages are given. Percentages in parentheses are calculated based on the non-missing (valid) N. MID = mild intellectual disability, MHD = mental health disorders, NL = The Netherlands, EU = Europe, N = total number of observations for each variable without missing.

*MID* = mild intellectual disability, *MHD* = mental health disorders, *NL* = The Netherlands, *EU* = Europe, *N* = total number of observations for each variable without missing

### Ethical approval and data linkage

This study was exempted from the Medical Research Involving Human Subjects Act by the Medical Ethics Committee (CEP number: N22.048). Routinely collected health data was securely stored, pseudonymized, and kept anonymous from researchers. Each participant received a unique record identification number (RIN) to ensure accurate linkage from three sources, preserving anonymity. Parents were linked to children via their RIN for family characteristics. SN staff verified output results to mitigate disclosure risks. Details on the linking procedure are available elsewhere [30].

### Variables

The dependent variable varied across analyses. For the first and third comparisons, it was the receipt of outpatient mental health care in a specialized center for children with MID, used as a proxy to identify children with both MID and MHD. For the second comparison, it was the receipt of outpatient mental health care, serving as a proxy to identify children with MHD. The independent variables encompassed various aspects across multiple domains.

#### Demographic domain

The demographic domain included child’s sex, age, birth country, and parental age and birth country. For both case groups, the child’s sex was determined from mental health care data. For the control groups, sex and age information was derived from SN, along with the birth country data of children and their parents for all groups. The use of country of birth as a substitute for ethnicity is widely accepted in Dutch literature [31].

#### Social and cultural domain

The social and cultural domain comprised four variables sourced from SN, each offering insight into various facets of the familial context. Firstly, the number of children in a household indicates the count of individuals, regardless of age or marital status, who share a child-parent relationship with one or both parents residing within a household. This encompasses biological, adopted, and stepchildren but excludes foster children. Secondly, family type, initially comprising eight categories, was later condensed into three: dual-parent households, single-parent households, and others, which encompass for example children from institutional households. Finally, maternal and paternal educational levels were examined, categorized as low, middle, or high based on the classification provided by SN. This categorization was determined by the highest level of education attained: primary education for low, vocational training for middle, and Higher Professional Education or university for high.

#### Economic domain

Standardized disposable household income was included, capturing the net annual amount available for spending, expressed in percentiles. This measure accounts for household size and composition. Disposable income was determined by subtracting a household’s total liabilities from its total assets. Relative disposable household income was based on population percentiles. Specifically, private households were categorized into 100 equal sized percentile groups, each defined by their standardized income. This approach ensured a fair and unbiased assessment of socioeconomic positions across the population, accounting for inflation.

#### Neighborhood domain

The neighborhood domain included three key factors, sourced from municipality data of The Hague. First, urbanization class was included based on average environmental address density (EAD) and categorized as densely (EAD ≥ 1500) and sparsely populated (EAD < 1500). EAD was computed as the number of addresses within a one-kilometer radius divided by the area. Second, “% low neighborhood education level” was included, representing the proportion of individuals aged 15 to 74 with lower educational attainment in neighborhoods within The Hague, determined by district codes (e.g., primary education, lower vocational education). Third, relative economic standing of a neighborhood, was measured by low median disposable income percentiles per postal code, adjusted for household size. Derived from standardized disposable income, this variable evaluates low median disposable income percentile within neighborhoods, ranging from the first to the twentieth percentile based on a national level. This score was adjusted for household size and composition within each postal code area.

### Approach to analysis

The analyses comprised several phases. First, descriptive statistics (*M* or %) for the SDOMH were calculated for all groups. Subsequently, univariate analyses were conducted to assess the individual association of each SDOMH with the presence of MID and MHD (Group A vs. Group C1). These analyses were not the focus of the study but served as a preliminary step to inform the multivariate models by identifying potential differences. Full multivariate logistic regression models then assessed associations between each SDOMH and the co-occurrence of MID and MHD (Group A vs. Group C1), adjusting for all other factors in the model. Further univariate and multivariate analyses examined the association between SDOMH and MHD without MID (Groups B vs. Group C2), and compared SDOMH for children with MHD with and without MID (Group A vs. Group B).

The “mice” (version 3.16.0), “modelsummary” (version 1.4.1), “forestplot” (version 3.1.1), and “stats” (version 4.3.1) packages for the R statistical software were used (v4.2.3) [32]. Unadjusted and adjusted odds ratios (*ORs*), 95% confidence intervals (CIs), and p-values (*p*) were calculated. We applied a significance level (*α*) of .05 to all statistical tests. Each OR represents the likelihood of a SDOMH being associated with undergoing mental health treatment for MHD in children with or without MID, compared to the control or between clinical groups. Multicollinearity among covariates was assessed using Pearson’s correlation coefficients and the variance inflation factor. The logistic regression model was then fitted using the glm function, with coefficients exponentiated to yield ORs. The ‘modelsummary’ package derived standardized ORs from our models, allowing comparison of the relative strength and direction of the relationships. Multiple imputations handled missing values, which ranged from 0% (child’s sex, age, birth country) to 50.4% (low median neighborhood income; see Table 1). Assuming data were missing at random, the Multivariate Imputation by Chained Equations method was used, which iteratively estimates each missing value through regression models conditional upon other variables. Twenty datasets were analyzed and pooled.

## Results

### Descriptive statistics

The study comprised 19,632 children (*M* = 10.6 years, *SD* = 3.2, range: 0 to 17), 35.6% of whom were female. Descriptive statistics of all the SDOMH are presented in Table 1 for each group. There were some differences in characteristics between the two case groups (Groups A and B). Children with MID receiving mental health care were significantly younger (see Fig. 3). Both case groups had a predominance of males, but children with MID receiving mental health care had the highest percentage, with 64.4% male.

### Key findings

**Fig. 1:**
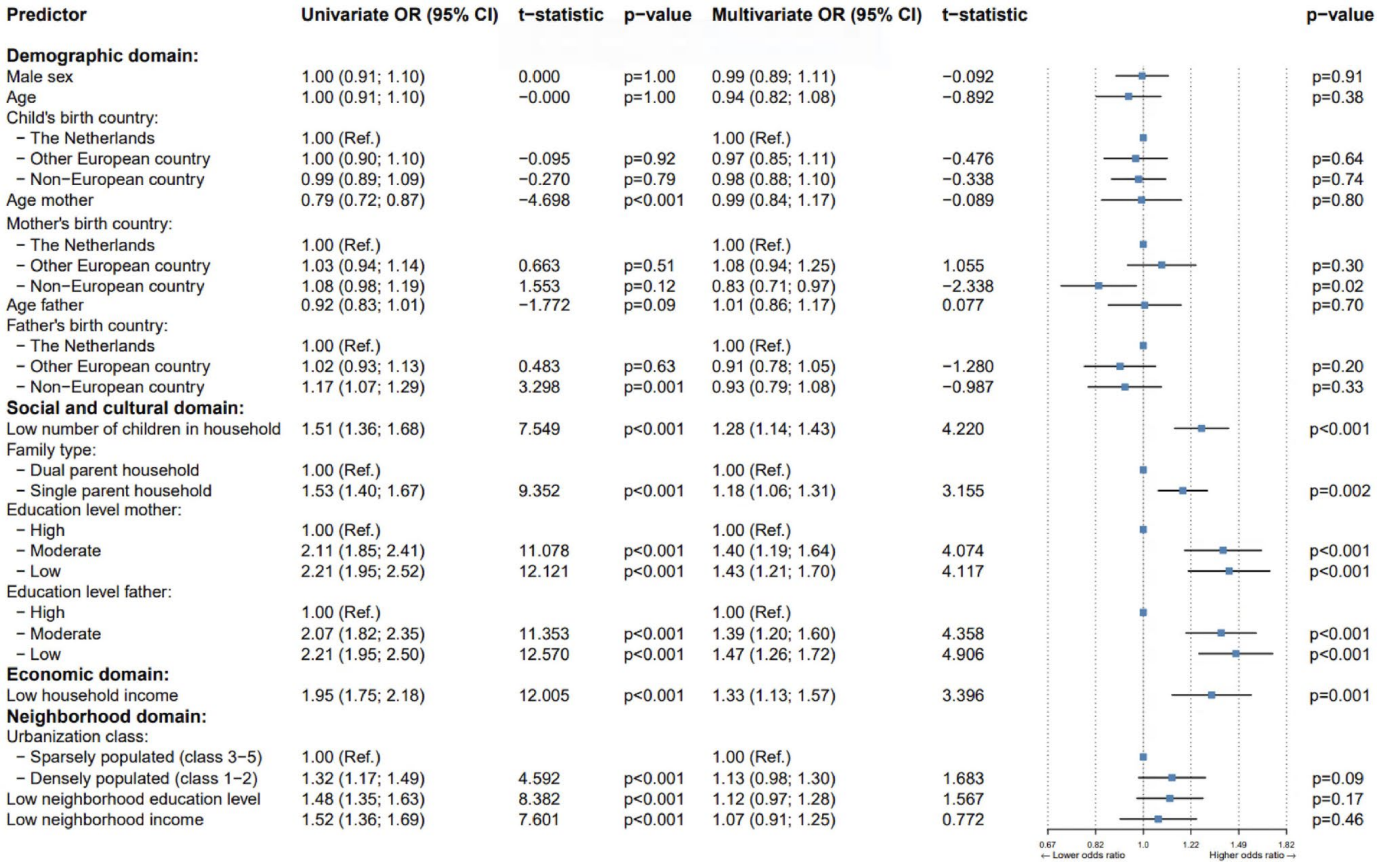
Forest plot of SDOMH among domains, comparing children having both ID and MHD (Group A) to their respective controls (Group C1). *OR* = odds ratio, *CI* = confidence interval. The error bars represent the 95% CI of the mean

In our first comparison, we compared children with MID receiving mental health care for MHD (referred to as MID + MHD, Group A) against their respective controls (Group C1). Figure 1 shows a forest plot presenting both univariate and multivariate ORs. Interpreting the multivariate results, our analysis revealed statistically significant associations between SDOMH and MID + MHD. Within the demographic domain, having a mother born outside Europe, was associated with lower odds of MID + MHD (*OR*: 0.83, *p* = .02). In contrast, the univariate analysis, which considers the variable in isolation, yielded slightly higher odds (*OR:* 1.08, *p* = .12) for children whose mothers were born outside Europe. Regarding the social and cultural domain, a lower number of children in a household was associated with higher odds for MID + MHD (*OR*: 1.28, *p* < .001). Living in a single-parent household was also associated with higher odds for MID + MHD (*OR*: 1.18, *p* = .002). Additionally, having a mother with a moderate (*OR*: 1.38, *p* < .001) or low education level (*OR*: 1.42, *p* = .001) was associated with higher odds of MID + MHD. A similar result was shown for the education level of fathers (moderate, *OR*: 1.40, *p* < .001; low, *OR*: 1.49, *p* < .001). In the economic domain, low household income was associated with MID + MHD (*OR*: 1.33, *p* = .001). Finally, in the neighborhood domain, no significant associations were observed between SDOMH and MID + MHD when compared to control children.

**Fig. 2:**
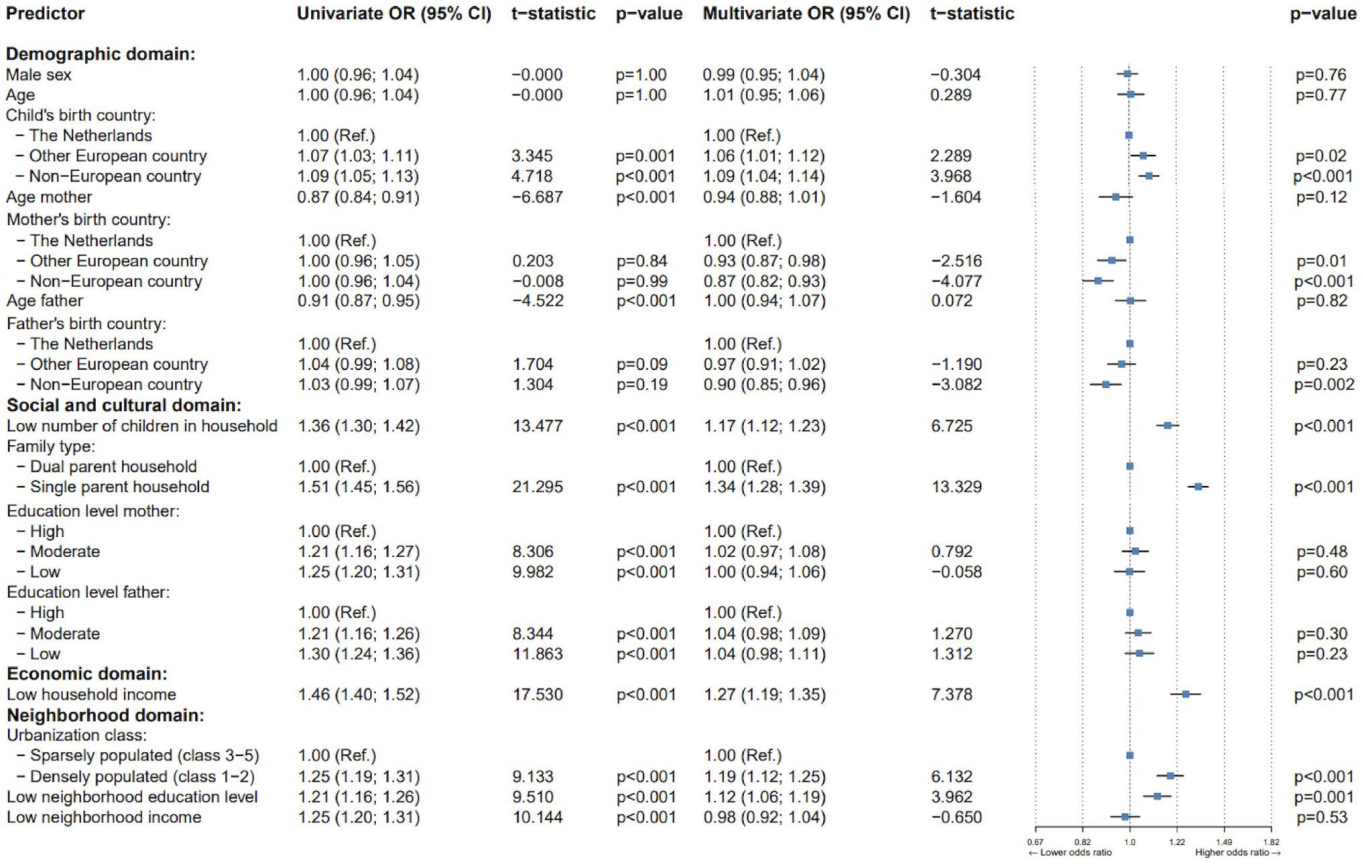
Forest plot of SDOMH among domains, comparing children having MHD (Group B) to their respective controls (Group C2). *OR* = odds ratio, *CI* = confidence interval. The error bars represent the 95% CI of the mean

In our second comparison, we compared children receiving mental health care for MHD, without MID (referred to MHD-only, Group B) to their respective controls (Group C2), as illustrated in Fig. 2. The following results will be based on multivariate ORs. In the demographic domain, the MHD-only group was more likely to be born outside the Netherlands within Europe (*OR*: 1.06, *p* = .02) or outside Europe (*OR*: 1.09, *p* < .001). Conversely, maternal birth outside the Netherlands, whether in Europe (*OR*: 0.93, *p* = .01) or outside Europe (*OR*: 0.87, *p* < .001), was associated with lower odds of MHD-only. Similarly, paternal birth outside Europe (*OR*: 0.90, *p* < .001) was linked to lower odds of MHD-only. In the social and cultural domain, a lower number of children in the household (*OR*: 1.17, *p* < .001) and single-parent households (*OR*: 1.34, *p* < .001) were associated with higher odds of MHD-only. In the economic domain, low household income (*OR*: 1.26, *p* < .001) was associated with MHD-only, aligning with the findings from the comparison between MID + MHD and controls. Regarding the neighborhood domain, residing in a densely populated urban area (*OR*: 1.19, *p* < .001) and areas with low neighborhood education levels (*OR*: 1.12, *p* = .001) were both linked to an increased OR for MHD-only.

**Fig. 3:**
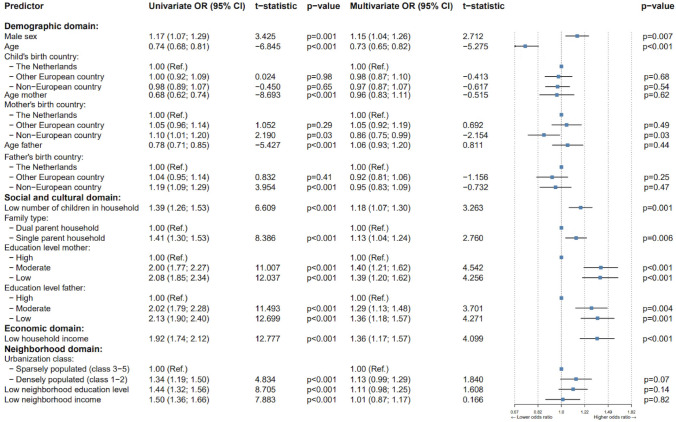
Forest plot of SDOMH among domains, comparing children having both ID and MHD (Group A) to those with MHD only (Group B). *OR* = Odds ratio, *CI* = confidence interval. The error bars represent the 95% CI of the mean

In our final comparison, we specifically focused on children receiving mental health care for their MHD, comparing children with and without MID (Groups A and B, respectively), as depicted in Fig. 3. Although the multivariate results were quite similar to those in Figs. 1 and 2, some differences were observed. Within the demographic domain, as these two groups had not been matched for age and sex, significant differences emerged. Being male (*OR*: 1.15, *p* = .008), along with a younger age (*OR*: 0.74; *p* < .001) were linked to higher odds of MID + MHD. Children with MID were less likely to have mothers born outside Europe compared to children without MID (*OR*: 0.86, *p* = .03). Within the social and cultural domain, MID + MHD tended to come from households with fewer children (*OR*: 1.18, *p* < .001) and were more likely to live in single-parent households (*OR*: 1.13, *p* = .006) compared MHD-only. Moreover, MID + MHD were significantly more likely to have parents with moderate (mother: *OR:* 1.38, *p* < .001; father: *OR:* 1.38, *p* = .001) or low education levels (mother: *OR:* 1.39, *p* < .001; father: *OR:* 1.39, *p* < .001). In the economic domain, households of children with MID displayed a significantly lower income (*OR*: 1.36, *p* < .001) compared to households of children without MID, mirroring the trends observed in previous comparisons. Within the neighborhood domain, no SDOMH was significantly linked to the likelihood of MID + MHD compared to MHD-only.

## Discussion

In this case–control study, we examined the associations between various SDOMH and MHD in children with MID. We compared children receiving mental health care with MID to their matched control group and to children without MID receiving mental health care. Additionally, we compared the latter group to their matched controls. By analyzing a comprehensive set of SDOMH across multiple domains, we aimed to identify those uniquely connected to children with MID undergoing mental health treatment. Our findings indicate that SDOMH across various domains are associated with receiving mental health care in children, irrespective of MID status. Children with MID face greater challenges, encountering more adverse SDOMH across multiple domains, which aligns with our hypothesis. Their disproportionate vulnerabilities emphasize the need for a comprehensive approach to mental health care that addresses the diverse challenges these children face. The study highlights three primary findings: the unique contributions of various domains of SDOMH, the significant effects of low parental education and household income, and the distinct neighborhood effects on children with and without MID.

As a first key finding, this study emphasizes the unique contribution of SDOMH in multiple domains, encompassing social, cultural, economic, and neighborhood contexts [17]. To the best of our knowledge, this study is the first to offer insight into how different SDOMH collectively play a role in receiving care for MHD among children with and without MID in the Netherlands. Our analyses revealed that the independent roles of several SDOMH across these domains remained significant, even when considered together in a multivariate model. Similarly, another study identified multiple determinants, such as lower SES and parental psychopathology, that were strongly associated with child behavioral problems in a multivariate model [33]. However, their study focused on children aged 9–11 without MID. These findings underscore the importance of recognizing the cumulative challenges faced by children with MHD and their families. Additionally, both these and our results highlight the need for a syndemic approach to address these complex healthcare needs. Such approach recognizes the interconnected nature of MHD and prioritizes integrated family care over symptom-focused interventions [34].

Our second key finding showed significant, independent links between low parental education levels and low household income with the likelihood of receiving mental health care, particularly among children with MID. Although parental education and income often correlate and are part of composite SES measures, these factors were particularly distinctive for children with MID. Regarding moderate and low parental education levels, these were only significantly more prevalent for children with MID. This association may be explained by several mechanisms. First of all, it aligns with prior research suggesting a connection between lower parental education and the likelihood of a child having an ID [35]. As lower parental education levels may be indicative of lower parental cognitive abilities, this association may be driven by the genetic hereditary of (M)ID [36]. Alternatively, in the framework of Lund et al. (2018), parental education level is viewed as an integral component of an individual’s social capital or network, which could be associated with the quantity and quality of social skills and support in the family [17]. Additionally, lower social support has been linked to increased parenting stress, lower child resilience, and poorer child mental health [37–39]. These factors support the double burden outlined in the introduction: children with MID face additional challenges due to a lack of resources, as a result their mental health may be more vulnerable to the adverse effects of SDOMH. Furthermore, contemporary society’s shift in Western countries towards individualism may present additional challenges, particularly for individuals with lower educational levels and resources. This shift emphasizes self-reliance over collective aid, exacerbating the difficulties faced by these children [40, 41].

Regarding lower household incomes, families with children receiving care for their MHD have lower household incomes compared to matched controls, regardless of whether the children have MID. However, households with children who have MID have the lowest incomes, as indicated by an OR just below the level associated with parental education. This aligns with previous research indicating that youth with MID often come from economically disadvantaged households compared to their peers without MID [8, 12, 42], supporting the first part of the double burden discussed in the introduction. The World Health Organization stated that MHD are closely linked to poverty, creating a cycle of systemic disadvantage [43]. This link may partially be explained by family processes of insecurity and cumulative stress [6, 44], while poverty is also associated with worse physical health status or stigma, which could play an additional role for MHD [17]. On a broader policy level, the high demand for mental health care among marginalized groups raises questions regarding the inclusivity of the Dutch society and the efficacy of preventive policies, particularly given the widening gap between the affluent and the underprivileged [45].

The third main finding is the difference in findings within the neighborhood domain for the two clinical case groups. Residing in areas with low neighborhood education levels and more densely populated areas was linked to an increased OR for children receiving care for MHD without MID, compared to the general population, but not for children with MID. This finding aligns with a systematic review showing that lower neighborhood SES was associated with increased problem behaviour in children without (M)ID [28]. To our knowledge, no studies have investigated neighborhood characteristics associated with the MHD in children with (M)ID. In our study, we did not observe significant multivariate effects for neighborhood characteristics in the MID + MHD group. However, we found that these children often lived in more densely populated, lower-income areas with lower levels of educational attainment. Future research should replicate these findings to better understand the role of neighborhood characteristics in MHD development in children with MID. Two potential explanations for the lack of significant neighborhood SDOMH include the comparatively smaller size of the MID + MHD group, which might result in limited statistical power, and a relatively greater importance of individual adversities for this group.

The study has several strengths. Firstly, it represents the first large-scale investigation conducted in the Netherlands examining children with MID. Our study leveraged extensive clinical and population-based samples, enabling a detailed analysis of SDOMH. This approach facilitated comparisons between children with and without MID receiving mental health care, enhancing the representativeness of our findings. Additionally, our study benefited from utilizing all available clinical data from a specialized mental health care center, ensuring diverse representation of children across various SES and ethnic backgrounds. Finally, our interpretation of multivariate results accounted for the independent effects of a comprehensive set of factors, providing a thorough understanding of the interconnected nature of SDOMH’s impact on mental health outcomes. This nuanced approach allowed us to identify the unique contributions of SDOMH on outcomes while controlling for potential confounders.

However, the results of this study might be interpreted in light of the following limitations. First, our study predominantly relied on data from a single institution specialized in care for children with MID and MHD. Although this institution is the largest in the area, the external validity of our findings remains uncertain. Consequently, it is necessary to validate our results using samples from other institutions, in more rural areas, and in different countries, as the roles of SDOMH can vary between societies. Additionally, we rely on data from children already engaged in outpatient mental health care, using service receipt as a proxy for identifying MHD. While this ensures a clinically validated approach, it excludes children with untreated mental health conditions and may underrepresent groups less likely to access these services, such as certain ethnic minorities [21, 46]. Furthermore, our reliance on real-world data led to a substantial number of missing data for some variables. Therefore, these results should be interpreted cautiously, particularly regarding low median neighborhood income, for which 50.4% of the data were missing. Another limitation is the reliance on cross-sectional data, restricting the ability to establish temporal and potential causal relationships. Finally, our selection of SDOMH was guided by literature from the general adult population without MID [17] and the availability of measurable variables. However, it remains uncertain whether other important SDOMH were omitted from this study, potentially limiting the comprehensiveness of our findings.

Taken together, our study underscores the profound impact of SDOMH on children with MHD and MID, revealing the unique and severe challenges they face. The findings advocate for a holistic, context-oriented approach to mental health care that addresses the diverse adversities experienced by these children. Moving forward, it is crucial for policymakers and practitioners to integrate comprehensive support for children and their family, alongside inclusive community strategies to reduce these systemic disadvantages, thereby creating a more equitable and supportive environment for all children.

## Data Availability

The study used data from the Extramural LUMC Academic Network data warehouse, a comprehensive regional population-based data infrastructure. Three primary data sources were analyzed: microdata from Statistics Netherlands (SN; the central register agency), The Hague municipality data, and patient data from specialized youth mental health care institutions. The data is not freely available due to privacy protections; it was pseudonymized and anonymized to prevent identification of individuals. This ensures confidentiality and minimizes disclosure risks.
